# Emerging local chikungunya virus transmission in a major urban area in Southern China: characteristics of clinical manifestations, viral evolution and climatic influences

**DOI:** 10.1093/nsr/nwaf529

**Published:** 2025-11-29

**Authors:** Jingyi Liang, Haisheng Yu, Zhengshi Lin, Xi Tang, Zhonghao Fang, Chitin Hon, Wenxin Hong, Lesi Kong, Yurou Wang, Yiwen Chen, Yuepeng Li, Yuelin Chen, Minling Guo, Shan Wu, Fengyu Hu, Haoran Qiu, Honglian Bai, Haiming Yan, Suhua Jiang, Qingsen Zhang, Jinfeng Liu, Huiling Zhou, Menglin Tan, Weijun Huang, Arlindo Oliveira, Jun Jiang, Yihui Huang, Zifeng Yang, Nanshan Zhong

**Affiliations:** Institute of Infectious Diseases, Guangzhou Eighth People’s Hospital, Guangzhou Medical University, Guangzhou 510440, China; State Key Laboratory of Respiratory Disease, National Clinical Research Center for Respiratory Disease, National Center for Respiratory Medicine, Joint International Research Laboratory of Respiratory Health, Guangdong Basic Research Center of Excellence for Respiratory Medicine, Guangzhou Institute of Respiratory Health, the First Affiliated Hospital of Guangzhou Medical University, Guangzhou 510120, China; China-Portugal Artificial Intelligence and Public Health Technologies Joint Laboratory, Guangdong-Hong Kong-Macao Joint Laboratory of Respiratory Infectious Diseases, Guangdong Provincial Key Laboratory of Respiratory Disease Research, Guangzhou Medical University, Guangzhou 510120, China; Institute of Infectious Diseases, Guangzhou Eighth People’s Hospital, Guangzhou Medical University, Guangzhou 510440, China; Guangzhou Key Laboratory of Clinical Pathogen Research for Infectious Diseases, Guangzhou Eighth People's Hospital, Guangzhou 510440, China; State Key Laboratory of Respiratory Disease, National Clinical Research Center for Respiratory Disease, National Center for Respiratory Medicine, Joint International Research Laboratory of Respiratory Health, Guangdong Basic Research Center of Excellence for Respiratory Medicine, Guangzhou Institute of Respiratory Health, the First Affiliated Hospital of Guangzhou Medical University, Guangzhou 510120, China; China-Portugal Artificial Intelligence and Public Health Technologies Joint Laboratory, Guangdong-Hong Kong-Macao Joint Laboratory of Respiratory Infectious Diseases, Guangdong Provincial Key Laboratory of Respiratory Disease Research, Guangzhou Medical University, Guangzhou 510120, China; Department of infectious disease, The First People’s Hospital of Foshan (Foshan Hospital Affiliated to Southern University of Science and Technology), Foshan 500062, China; State Key Laboratory of Respiratory Disease, National Clinical Research Center for Respiratory Disease, National Center for Respiratory Medicine, Joint International Research Laboratory of Respiratory Health, Guangdong Basic Research Center of Excellence for Respiratory Medicine, Guangzhou Institute of Respiratory Health, the First Affiliated Hospital of Guangzhou Medical University, Guangzhou 510120, China; Department of Engineering Science, Faculty of innovation Engineering, Macau University of Science and Technology, Taipa, Macau 999078, China; Respiratory Disease AI Laboratory on Epidemic Intelligence and Medical Big Data Instrument Applications, Macau University of Science and Technology, Macau 999078, China; Institute of Infectious Diseases, Guangzhou Eighth People’s Hospital, Guangzhou Medical University, Guangzhou 510440, China; State Key Laboratory of Respiratory Disease, National Clinical Research Center for Respiratory Disease, National Center for Respiratory Medicine, Joint International Research Laboratory of Respiratory Health, Guangdong Basic Research Center of Excellence for Respiratory Medicine, Guangzhou Institute of Respiratory Health, the First Affiliated Hospital of Guangzhou Medical University, Guangzhou 510120, China; Institute of Infectious Diseases, Guangzhou Eighth People’s Hospital, Guangzhou Medical University, Guangzhou 510440, China; Guangzhou Key Laboratory of Clinical Pathogen Research for Infectious Diseases, Guangzhou Eighth People's Hospital, Guangzhou 510440, China; Institute of Infectious Diseases, Guangzhou Eighth People’s Hospital, Guangzhou Medical University, Guangzhou 510440, China; School of Public Health, Sun Yat-sen University, Guangzhou 510275, China; State Key Laboratory of Respiratory Disease, National Clinical Research Center for Respiratory Disease, National Center for Respiratory Medicine, Joint International Research Laboratory of Respiratory Health, Guangdong Basic Research Center of Excellence for Respiratory Medicine, Guangzhou Institute of Respiratory Health, the First Affiliated Hospital of Guangzhou Medical University, Guangzhou 510120, China; State Key Laboratory of Respiratory Disease, National Clinical Research Center for Respiratory Disease, National Center for Respiratory Medicine, Joint International Research Laboratory of Respiratory Health, Guangdong Basic Research Center of Excellence for Respiratory Medicine, Guangzhou Institute of Respiratory Health, the First Affiliated Hospital of Guangzhou Medical University, Guangzhou 510120, China; Institute of Infectious Diseases, Guangzhou Eighth People’s Hospital, Guangzhou Medical University, Guangzhou 510440, China; Guangzhou Key Laboratory of Clinical Pathogen Research for Infectious Diseases, Guangzhou Eighth People's Hospital, Guangzhou 510440, China; Institute of Infectious Diseases, Guangzhou Eighth People’s Hospital, Guangzhou Medical University, Guangzhou 510440, China; Guangzhou Key Laboratory of Clinical Pathogen Research for Infectious Diseases, Guangzhou Eighth People's Hospital, Guangzhou 510440, China; Institute of Infectious Diseases, Guangzhou Eighth People’s Hospital, Guangzhou Medical University, Guangzhou 510440, China; Guangzhou Key Laboratory of Clinical Pathogen Research for Infectious Diseases, Guangzhou Eighth People's Hospital, Guangzhou 510440, China; State Key Laboratory of Respiratory Disease, National Clinical Research Center for Respiratory Disease, National Center for Respiratory Medicine, Joint International Research Laboratory of Respiratory Health, Guangdong Basic Research Center of Excellence for Respiratory Medicine, Guangzhou Institute of Respiratory Health, the First Affiliated Hospital of Guangzhou Medical University, Guangzhou 510120, China; Department of infectious disease, The First People’s Hospital of Foshan (Foshan Hospital Affiliated to Southern University of Science and Technology), Foshan 500062, China; Department of infectious disease, The First People’s Hospital of Foshan (Foshan Hospital Affiliated to Southern University of Science and Technology), Foshan 500062, China; Department of infectious disease, The First People’s Hospital of Foshan (Foshan Hospital Affiliated to Southern University of Science and Technology), Foshan 500062, China; Department of infectious disease, The First People’s Hospital of Foshan (Foshan Hospital Affiliated to Southern University of Science and Technology), Foshan 500062, China; Department of infectious disease, The First People’s Hospital of Foshan (Foshan Hospital Affiliated to Southern University of Science and Technology), Foshan 500062, China; Department of infectious disease, The First People’s Hospital of Foshan (Foshan Hospital Affiliated to Southern University of Science and Technology), Foshan 500062, China; Department of infectious disease, The First People’s Hospital of Foshan (Foshan Hospital Affiliated to Southern University of Science and Technology), Foshan 500062, China; Department of infectious disease, The First People’s Hospital of Foshan (Foshan Hospital Affiliated to Southern University of Science and Technology), Foshan 500062, China; INESC-ID, Instituto Superior Técnico, Universidade de Lisboa, 1049-001 Lisbon, Portugal; Department of infectious disease, The First People’s Hospital of Foshan (Foshan Hospital Affiliated to Southern University of Science and Technology), Foshan 500062, China; Institute of Infectious Diseases, Guangzhou Eighth People’s Hospital, Guangzhou Medical University, Guangzhou 510440, China; State Key Laboratory of Respiratory Disease, National Clinical Research Center for Respiratory Disease, National Center for Respiratory Medicine, Joint International Research Laboratory of Respiratory Health, Guangdong Basic Research Center of Excellence for Respiratory Medicine, Guangzhou Institute of Respiratory Health, the First Affiliated Hospital of Guangzhou Medical University, Guangzhou 510120, China; China-Portugal Artificial Intelligence and Public Health Technologies Joint Laboratory, Guangdong-Hong Kong-Macao Joint Laboratory of Respiratory Infectious Diseases, Guangdong Provincial Key Laboratory of Respiratory Disease Research, Guangzhou Medical University, Guangzhou 510120, China; Guangzhou Laboratory, Bio-Island, Guangzhou 510320, China; State Key Laboratory of Respiratory Disease, National Clinical Research Center for Respiratory Disease, National Center for Respiratory Medicine, Joint International Research Laboratory of Respiratory Health, Guangdong Basic Research Center of Excellence for Respiratory Medicine, Guangzhou Institute of Respiratory Health, the First Affiliated Hospital of Guangzhou Medical University, Guangzhou 510120, China; China-Portugal Artificial Intelligence and Public Health Technologies Joint Laboratory, Guangdong-Hong Kong-Macao Joint Laboratory of Respiratory Infectious Diseases, Guangdong Provincial Key Laboratory of Respiratory Disease Research, Guangzhou Medical University, Guangzhou 510120, China; Guangzhou Laboratory, Bio-Island, Guangzhou 510320, China

**Keywords:** chikungunya fever, clinical characteristic, virus, transmission, climate

## Abstract

The 2025 chikungunya fever outbreak in Foshan, China rapidly spread from a previously non-endemic area, raising significant public health concerns. This event underscores the need to understand the factors driving viral transmission, host responses and the influence of local environmental changes. The primary objectives of this study were to: (i) characterize the clinical manifestations of chikungunya patients at the initial stage of the outbreak to facilitate concise diagnosis and treatment; (ii) determine the viral genetic factors contributing to the outbreak’s rapid spread; and (iii) investigate the influence of local environmental and climatic conditions on vector mosquito reproduction. We quickly collected and analyzed clinical data from 134 patients hospitalized for the purpose of quarantine at the beginning of the outbreak. While fever, arthralgia and rash are the typical symptom triad of chikungunya fever, we found that they did not always present simultaneously at onset. Arthralgia was the most common presenting symptom. Phylogenetic analysis revealed that the viral strains were highly homologous to those from the Réunion outbreak, suggesting an imported origin. Furthermore, we identified the presence of E1-A226V, E2-I211T and E2-L210Q mutations, which have been previously associated with enhanced transmission by *Aedes albopictus*. Local climatic conditions during the outbreak period were also found to be favorable for mosquito reproduction. In conclusion, we propose that the Foshan outbreak resulted from a combination of virus importation, a largely immunologically naïve population and a climate conducive to mosquito proliferation. Additionally, our findings suggest that clinicians should maintain vigilance for atypical symptoms to prevent misdiagnosis and missed cases.

## INTRODUCTION

A chikungunya fever (CHIKF) outbreak has been occurring in Foshan, China since early July 2025 [1]. As of 8 August 2025, there has been a rapid increase of over 8000 reported cases [2]. Since 2008, China has experienced several CHIKF outbreaks in tropical and subtropical regions [3,4]. However, this sudden outbreak in Foshan, where

the virus was previously absent, is unprecedented. This situation prompts us to conduct an in-depth study of the outbreak’s epidemiological characteristics and clinical features to facilitate the rapid implementation of control measures.

CHIKF is a vector-borne disease caused by the chikungunya virus (CHIKV), which is primarily transmitted to people through the bite of an infected mosquito, mainly *Aedes aegypti* and *Aedes albopictus* [5–8]. CHIKV belongs to the family Togaviridae and the genus *Alphavirus* [8]. CHIKV is a single-stranded positive-sense RNA virus, comprising one serotype and three genotypes: West African, East/Central/South African (ECSA) and Asian, with ECSA and Asian genotypes being the predominant circulating strains [9]. The typical clinical presentation of CHIKF is characterized by sudden onset of fever, severe joint pain and swelling (often in the small joints of the hands and feet), and a maculopapular rash. Additional symptoms may include headache, muscle pain and fatigue, but they rarely cause death. The disease is usually mild; however, its articular symptoms may progress over months into chronic arthritis [10]. Currently, no specific antiviral treatment exists, and clinical management focuses on supportive care and symptom control.

Foshan is a city with a population of 9.5 million in Guangdong Province, China, located in the heart of the Pearl River Delta. Not only does Foshan have a mild climate (average annual temperature of 23.2°C) and year-round abundant rainfall, but it is also a vital manufacturing hub and has well-developed commerce and tourism industries. According to the statistics from the World Health Organization (WHO), documented cases of local CHIKV transmission so far have been reported in 119 countries and regions, primarily in tropical and subtropical areas such as South America, Africa, and Southeast Asia and Indian Ocean islands, causing 1.95 million disability-adjusted life years globally between 2011 and 2020 [11].

Globalization, climate change and urbanization have clearly facilitated arboviral disease transmission, as evidenced by a significant shift in the transmission patterns of CHIKV [12]. In the context of CHIKF outbreaks, the One Health framework is particularly pertinent [13]. The ongoing global spread of CHIKV highlights the interconnection between human health and environmental conditions, which presents significant challenges to global public health governance systems.

While Foshan has implemented multifaceted preventive measures to contain CHIKV transmission, some issues remain for further investigation, particularly regarding the origin of the virus in the Foshan outbreak, the influence of local climate change on mosquito reproduction, and the increase in doctors’ diagnostic accuracy based on patients’ initial symptoms in a new epidemic area. Through an in-depth analysis of clinical data from patients with CHIKF in Foshan, along with a thorough examination of the virological features of CHIKV, we identified that the outbreak strain originated from Réunion. Remarkably, the clinical presentation of the illness was relatively mild; the simultaneous occurrence of the classic symptom triad—fever, rash and arthralgia—was uncommon as presenting symptoms, with a significant number of patients reporting only arthralgia as their first symptom, and these symptoms varied across different age groups. This evidence underscores the significance of understanding the unique aspects of CHIKV infection in Foshan, highlighting the need for ongoing research and tailored public health strategies to address this challenge effectively.

## RESULTS

**Figure 1.**
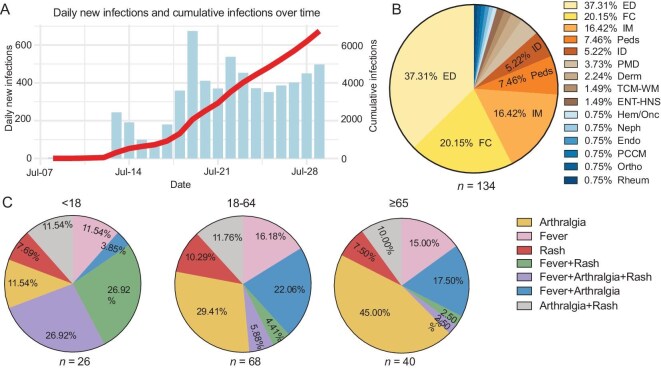
The CHIKF outbreak in Foshan. (A) Temporal progression of the infection over time; (B) the proportions of the clinical departments that patients first visited. (C) Distribution of the initial symptom combinations among chikungunya virus cases across different age groups. ED, Emergency Department; FC, Fever Clinic; IM, Internal Medicine; Peds, Pediatrics; ID, Infectious Disease; PMD, Primary Medical Department; Derm, Dermatology; TCM-WM, Traditional Chinese and Western Medicine; ENT-HNS, Ear, Nose, Throat—Head and Neck Surgery; Hem/Onc, Hematology/Oncology; Neph, Nephrology; Endo, Endocrinology; PCCM, Pulmonary and Critical Care Medicine; Ortho, Orthopedics; Rheum, Rheumatology.

The CHIKF outbreak in Foshan City began on 9 July 2025, with Shunde District identified as the initial epicenter. The outbreak subsequently disseminated to adjacent districts. As shown in Fig. 1A, the number of daily new infections escalated sharply from 13 July onward, peaking around 18 July with over 650 cases reported in a single day. As of 1 August, a cumulative total of 6300 confirmed infections had been reported. The basic reproduction number (R0) was 3.50 [95% confidential interval (CI): 1.50–4.12].

Since the scale of the CHIKF outbreak in Foshan is unprecedented in China, it is essential to conduct an in-depth study on its clinical characteristics to provide an accurate basis for clinical diagnosis and the formulation of public health prevention measures. We focused on 134 CHIKF cases admitted to the First People’s Hospital of Foshan, and the diagnosis was confirmed by real‐time polymerase chain reaction (RT-PCR). The patients’ residential areas were distributed across several neighboring districts, with 73 (54.78%) from the Shunde District, 45 from the Chancheng District, 15 from the Nancheng District and 1 from the Sanshui District. Patients’ average age was 49 years, and 19.40% (*n* = 26) were <18 years old; 51.90% were male. Among these patients, 33 had hypertension, 27 had coronary heart disease, 8 had diabetes and 2 were pregnant. Ten cases were identified as a clustering infection from five households (Table 1).

**Table 1.** Demographic characteristic of hospitalized patients.

	Overall	<18 years	18–64 years	≥65 years	
Characteristic	(*N* = 134)	(*n* = 26)	(*n* = 68)	(*n* = 40)	*P* value
Male, *n* (%)	70 (52.2)	17 (65.4)	27 (39.7)	26 (65.0)	0.013*
Age *n* (median[P_25_–P_75_])	49.0	10.5	45.0	75.0	
	[27.8–67.5]	[6.8–13.0]	[32.3–54.0]	[70.0–80.0]	
Comorbidity					
Hypertension *n* (%)	33 (24.6)	0	8 (11.8)	25 (62.5)	<0.001**
Coronary heart disease *n* (%)	7 (5.4)	0	0	7 (17.5)	<0.001**
Diabetes *n* (%)	9 (6.7)	0	3 (4.4)	6 (15.0)	0.033*
Pregnancy *n*	2	0	2	0	
Clustering cases *n*	10	1	8	1	

Data are presented as case number (percentage %], median [P_25_–P_75_]. Group comparisons were performed using the Chi-square test for categorical variables and the Kruskal-Wallis test for continuous variables. * : *P* < 0.05, **: *P* < 0.01 was considered significant.

The patients in our cohort did not always exhibit the typical symptom triad of CHIKF—fever, arthralgia and skin rash, as the initial clinical presentations at the same time; therefore, their initial hospital visits ended up in different departments: emergency department (37.31%); fever clinic (20.15%); and internal medicine (16.15%). Interestingly, there were small proportions of the patients who first visited the departments of pain management (3.73%), dermatology (2.24%) or orthopedics (0.75%) (Fig. 1B), indicating existence of atypical onset of the illness. On further analysis of the initial symptoms, we observed that the onset of the symptoms—fever, rash and arthralgia—was in different combinations across various age groups (Fig. 1C). Among them, arthralgia as a single initial and the most common symptom occurred in 11.54% of the minor group (<18 years), 29.41% of the adult group (18–64 years) and 45.00% of the elderly group (≥ 65 years). Fever as the initial symptom occurred in only 11.54% of the minor group, 16.80% of the adult group and 15% of the elderly group. Rash as the initial symptom occurred less frequently than arthralgia and fever, ranging from 7.69% of the minor group, 10.29% of the adult group and 7.51% of the elderly group. Interestingly, the classic symptom triad of CHIKF in this cohort occurred at the same time most frequently in the minor group (26.92%), compared to the adult group (5.88%) and the elderly group (2.50%). These age-related differences in the initial clinical manifestations of the disease should raise an alert for the front-line clinicians.

The patterns of the initial symptom presentation did not carry over to the later stage of the illness. Overall, as shown in Table 2, this cohort eventually had 90.3% (*n* = 121) of patients developing a fever, 46.3% (*n* = 62) with a moderate fever (38.1°C–39.0°C); the fever lasted for about 2 days on average across all age groups. Arthralgia occurred in 88.8% (*n* = 119) of the patients, and lasted 1 to 3 days on average in different age groups, with the most extended duration in the 18–64 years group (*P* = 0.001). Maculopapular rash occurred in 82.1% of the patients; the minor group (<18 years) had the longest time of rash presentation (*P* < 0.001). Blood tests in all age groups were within normal ranges, as well as the inflammatory indicators (Table S2). Age, sex and chronic comorbidities (hypertension, coronary heart disease and diabetes) did not exert a significant influence on the occurrence of atypical clinical manifestations in this cohort (Table S3).

**Table 2.** Comparison of clinical manifestation and laboratory testing in different age groups.

	Overall	<18 years	18–64 years	≥65 years	
	(*N* = 134)	(*n* = 26)	(*n* = 68)	(*n* = 40)	*P* value
Fever *n* (%)	121 (90.3)	24 (92.3)	62 (91.2)	35 (87.5)	0.764
Low *n* (%)	39 (29.1)	7 (26.9)	24 (35.3)	8 (20.0)	<0.001**
Moderate *n* (%)	62 (46.3)	9 (34.6)	32 (47.1)	21 (52.5)	0.001**
High *n* (%)	20 (14.9)	8 (30.8)	6 (8.8)	6 (15)	0.886
Rash (%)	110 (82.1)	24 (92.3)	56 (82.4)	30 (75.0)	0.200
Whole body (%)	30 (22.4%)	2 (7.7%)	25 (36.8%)	3 (7.5%)	
Trunk (%)	61 (45.5%)	19 (73.1%)	22 (32.4%)	20 (50.0%)	
Limbs (%)	63 (47.0%)	24 (92.3%)	25 (36.8%)	14 (35.0%)	
Face (%)	23 (17.2%)	13 (50%)	5 (7.4%)	5 (12.5%)	
Arthralgia (%)	119 (88.8)	15 (57.7)	66 (97.1)	38 (95.0)	<0.001**
Score of joint pain [Median (IQR)]					
No pain	15 (11.2%)	11 (42.3%)	2 (2.9%)	2 (5.0%)	
Mild pain	64 (47.8%)	12 (46.2%)	34 (50.0%)	18 (45.0%)	
Moderate pain	42 (31.3%)	2 (7.7%)	24 (35.3%)	16 (40.0%)	
Severe pain	13 (9.7%)	1 (3.8%)	8 (11.8%)	4 (10.0%)	
Headache (%)	44 (32.3)	7 (24.0)	28 (41.2)	9 (22.5)	0.886
Myalgia (%)	19 (14.2)	2 (7.7)	10 (14.7)	7 (17.5)	0.528
Asthenia (%)	48 (35.8)	3 (11.5)	27 (39.7)	18 (45.0)	0.014*
Conjunctival congestion (%)	26 (19.4)	3 (11.5)	16 (23.5)	7 (17.5)	0.394
Erythema of pectoral muscles (%)	30 (22.6)	7 (28.0)	15 (22.1)	8 (20.0)	0.747
Nausea/vomiting (%)	15 (11.2)	5 (19.2)	4 (5.9)	6 (15.0)	0.122
Lymphadenopathy (%)	34 (25.4)	7 (26.9)	19 (27.9)	8 (20.0)	0.644
Synovial hyperplasia (%)	32 (32.7)	3 (20.0)	19 (38.8)	10 (29.4)	0.352
Joint effusion (%)	63 (64.3)	8 (53.3)	32 (65.3)	23 (67.6)	0.615
Length of hospital stay	5.0[4.0–5.0]	5.0[5.0–6.0]	4.0[3.0–5.0]	5.0[5.0–6.0]	<0.001**
Duration of symptom					
Fever	2.0[1.0–2.0]	2.0[1.8–3.0]	2.0[1.0–2.0]	2.0[1.0–2.0]	0.189
Rash	3.0[2.0–4.0]	6.0[3.8–6.0]	2.0[2.0–3.0]	2.0[0.5–3.0]	<0.001**
Arthralgia	2.0[2.0–3.0]	1.0[0.0–3.0]	3.0[2.0–3.0]	2.0[2.0–3.0]	0.001**

Data are presented as case number (percentage %], median [P_25_–P_75_]. Group comparisons were performed using the Chi-square test for categorical variables and the Kruskal-Wallis test for continuous variables. * :*P* < 0.05, **: *P* < 0.01 was considered significant.

### Fever characteristics

**Figure 2.**
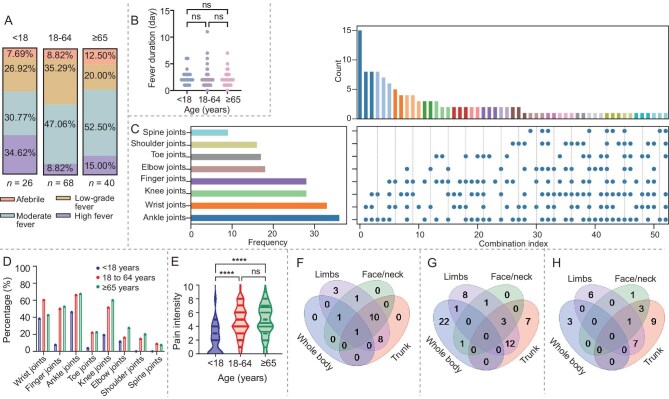
Characteristic of fever, joint pain and rash of Foshan CHIKF patients. (A) Fever patterns among CHIKF patients across different age groups. Stacked bar charts illustrating the proportion of patients with afebrile presentation (pink), low-grade fever (brown), moderate fever (dark blue) and high fever (purple). Body temperature was measured as the patient’s axillary temperature. Fever classification was defined as afebrile (≤37.2°C), low fever (37.3°C–38.0°C), moderate fever (38.1°C–39.0°C) or high fever (39.1°C–40.0°C). (B) Dot plot of fever duration across age groups. (C) Characteristics of the affected joints among the CHIKF patients. UpSet plot showing the frequency and combinations of affected joints. (D) The percentage of the joints involved across different age groups. (E) Distribution of joint pain scores (assessed with age-appropriate scales: NRS for ≥5 years, FPS-R for <5 years) across age groups. ****P < 0.0001; ns, not significant. Venn diagrams showing anatomical distribution patterns of rash among chikungunya patients in different age groups: (F) <18 years old, *n* = 24; (G) 18–64 years old, *n* = 56; (H) ≥65 years old, *n* = 30.

Among the patients with a fever, the minors were more prone to high fever. In contrast, adults predominantly exhibited a moderate fever (Table 2, Fig. 2A). No significant correlation was observed between fever duration and age (Fig. 2B). Although the individual body temperature variations were significant, the duration of fever was similar across all age groups.

### Characteristics of the joint involvement

In the study population, the most frequently affected joints were, in order, the ankle (74.6%), wrist (70.1%), knee (61.2%) and finger joints (58.2%), followed by the elbow (20.1%), toe joints (17.2%), shoulder (9.0%) and spine (3.7%). The UpSet plot (Fig. 2C) revealed that combined involvement of the ankle and wrist joints was the most common, with complex patterns of polyarticular involvement, and a high proportion of cases involving three or more joints. It is worth noting that 3.7% of adult patients and 42.31% of minors did not present arthralgia at all. Further age subgroup analysis showed that adult patients (>18 years old, *n* = 108) exhibited more extensive joint involvement patterns compared to minors (<18 years old, *n* = 26; Fig. 2D). Specifically, the ankle, finger, toe and knee joint involvement was significantly more prevalent in the adult group than in the minor group, indicating that pediatric patients generally less commonly had joint involvement. Using a 10-point scale to assess joint pain severity, the median score in the <18-year-old group was approximately 2–3, while the 18–64-year-old and ≥65-year-old groups both had median scores of around 5–6 (Fig. 2E); the scores in the the adult and elderly groups were significantly higher than those in the <18-year-old group (*P* < 0.0001), whereas no statistically significant difference was found between the 18–64-year-old and ≥65-year-old groups (*P* ≥ 0.05). Figure S1 shows the distribution of various scores in the adult and minor groups. In summary, the CHIKF patients in this cohort most commonly experienced joint pain in the ankles, wrists, knees and finger joints, with most cases exhibiting polyarticular involvement. Significant differences existed across various age groups in terms of the number of joints involved and the pain intensity.

### Rash

Not all CHIKF patients developed skin rash; 64.2% (*n* = 86) of adult patients and 17.9% (*n* = 24) of patients <18 years old presented a skin rash in the early stage of the disease. A typical rash of CHIKV infection from one adult patient is shown in Fig. S2: on Day 1 (initial stage), the rash was sparse, more localized and symmetric, and often seen in the upper limbs and abdominal area. It was maculopapular, with small, red, flat/raised spots that appeared not fully developed yet. The rash had become more pronounced and widespread on Day 2 (progression stage), particularly on the chest and upper body. This pattern is typical of CHIKF viral exanthema. On Day 3 (advanced stage), the maculopapular rash remained, spreading further to the lower body, including the legs, and became more confluent, with some areas showing more intense redness.

The skin rash in the adult group was the most widespread, with simultaneous involvement of the trunk, limbs and face/neck; over 30% had whole-body coverage (Fig. 2G). In contrast, children under 18 years old showed more localized rash patterns, most commonly limited to the limbs, with only a small number of the patients exhibiting multiregional distribution (Fig. 2F). The extent of the rash on the elderly patients (≥65 years) was between the other two groups, with trunk and limb involvement being most prevalent (Fig. 2H). However, whole-body involvement was relatively rare. Similar to the arthralgia manifestation, the rash manifestation in the adult patients was more severe compared to the other two age groups.

### Viral loads

**Figure 3.**
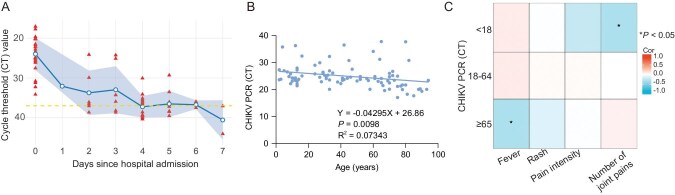
Viral load dynamics and age-related correlation in chikungunya patients. (A) The blue line represents the daily median CT value, with shaded areas indicating IQRs. The red triangles represent patients’ CT values. The yellow dashed line at CT = 38 marks the diagnostic threshold for viral negativity. (B) Correlation between baseline CT values and the patients’ ages. (C) Age-stratified correlations between viral cycle threshold values and clinical symptoms (fever, rash, pain intensity and number of painful joints).

The CHIK viral loads [cycle threshold (CT) values] from the patients’ serum gradually declined during hospitalization, with the majority of patients achieving viral clearance by Days 6–7 (Fig. 3A), indicating relatively rapid viral elimination. The initial viral loads and age were somewhat positively correlated (Fig. 3B; *P* = 0.0098). This weak association might be due to individual variability in immune responses. On the other hand, the timing of specimen collection varied because not all patients sought medical care immediately upon symptom onset; a relatively small sample size might also be another factor. Although the correlation was weak, it might suggest that the older patients might have higher viral loads at the initial stage of the disease. We have performed additional age-stratified analyses to examine the correlation between viral load and clinical manifestations. In pediatric patients (<18 years old), viral load was positively correlated with the number of painful joints, indicating that higher viral load was associated with more extensive joint involvement. In elderly patients (>65 years old), viral load was positively correlated with peak body temperature (Fig. 3C). These findings highlight age-specific differences in the positive relationship between viral load and symptom severity.

### Phylogenetic and amino acid variation analysis

**Figure 4.**
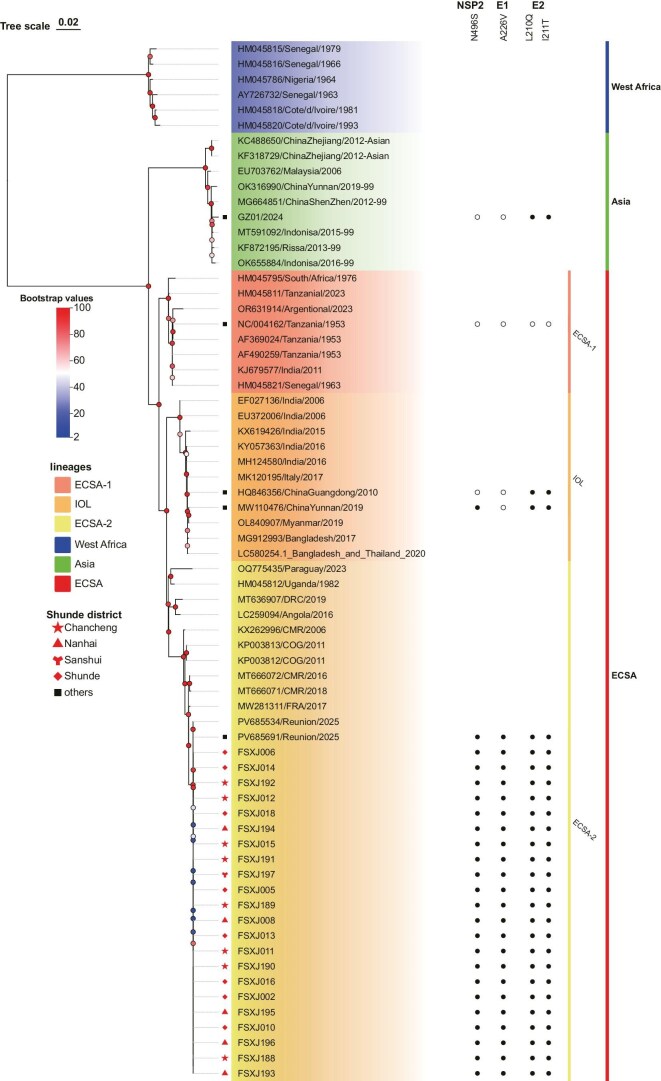
Maximum-likelihood phylogenetic reconstruction was performed using IQ-TREE with the best-fitting nucleotide substitution model and 1000 ultrafast bootstrap replicates. Filled red circles represent the 22 samples included in the study, filled black squares represent strains used for mutation analysis, and red pentagrams indicate strains from previous domestic chikungunya outbreaks. Different-colored blocks correspond to different lineages, with the expansion value shown as a heatmap. Full spheres next to the tree leaves indicate the presence of specific mutations in the E1, E2 or NSP2 genes.

One critical question is whether the current Foshan CHIKF outbreak was caused by imported virus through travelers or by strains that have previously caused sporadic endemics in China. We compared the evolutionary relationship of the genomes of the 22 Foshan CHIKV strains (FSX-) with the other 46 chikungunya viral strains reported previously. The phylogenetic tree revealed that the Foshan CHIKV strains are highly homogeneous with PV685691/Réunion/2025 and PV685534/Réunion/2025 most recently isolated from the patients in Réunion with a 100% bootstrap value, which all belong to the ECSA-2 lineage (Fig. 4). Previously identified CHIKV strains in China that caused sporadic CHIKF cases, the Guangzhou strain (GZ01/2024), the two Zhejiang strains (KC488650, KF318729), the Yunnan strain (OK316990) and the Shenzhen strain (MG664851) are all clustered in the Asian genotype (bootstrap value 90%). Additionally, the 2019 Yunnan strain (MW110476) belongs to the IOL genotype (bootstrap value 100%). This evidence suggests that the circulating Foshan CHIKV strains originated from the imported virus, rather than from strains previously found in China.

We further specifically compared the amino acid sequences of the 22 Foshan-CHIKV strains with some of those of the CHIKV strains isolated from China and Réunion: the GZ01/2024 (Asian genotype); HQ846356/ChinaGuangdong/2010 (IOL genotype); MW110476/ChinaYunnan/2019 (IOL genotype); and PV685691/Réunion/2025 (ECSA-2 genotype) against the default NCBI reference strain NC004162/Tanzania/1953 (Table S1). The amino acid sequences of the 22 Foshan-CHIKV strains were identical to PV685691 associated with the Réunion epidemic in 2024–25, except for the FSXJ015 strain, which had alanine at position 263 of NSP4 instead of proline in other Foshan strains. The Foshan strains carried the mutation NSP2-N495S, which has been suggested to affect the substrate recognition function of the protein [14]. Moreover, previously mentioned combination mutations of E1-A226V, E2-I211T and E2-L210Q, associated with the Réunion lineage, were also present in Foshan strains. These mutations could enhance the transmission ability of CHIKV in *A. albopictus* [15], potentially promoting local virus spread and possibly contributing to the current Foshan outbreak of chikungunya.

### Local climate change favors mosquito reproduction

**Figure 5.**
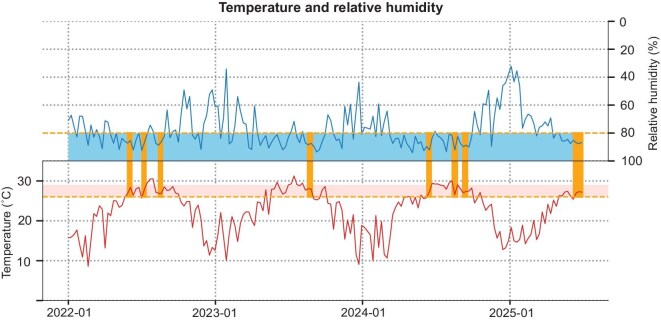
Weekly temperature and relative humidity trends with climatic suitability windows (2022–25). Time-series visualization of weekly temperature and relative humidity in Foshan, China from January 2022 to July 2025, with identification of climatic periods conducive to vector-borne disease transmission. The upper panel displays relative humidity (%) (blue dashed line, right *y*-axis, inverted), with the high-humidity zone (≥80%) shaded in light blue. The lower panel presents temperature (°C) (red line, left *y*-axis), with the optimal thermal range (26°C–29°C) highlighted in light red. Overlapping periods in which both optimal temperature and relative humidity were simultaneously met for 10 or more consecutive days are shaded in light orange across both panels.

In addition to studying the origin of the CHIKV strain leading to this outbreak and the viral mutation, we further investigated whether Foshan’s climatic conditions were conducive to the spread of the epidemic. We observed that the number of cumulative days with climate (temperature and humidity) suitable for mosquito reproduction from 2021 to 2024 increased year by year. However, the most extended period, 38 days in a row, was in 2025, just before July, the month of the CHIKF outbreak (Fig. 5). Additionally, a continuous 23-day peak optimal period was significantly longer than that observed in 2022–24 (Table S4). In summary, the climate change in Foshan in 2025 may favor mosquito population growth, thus increasing the risk of vector-borne disease transmission.

Furthermore, we examined the temporal associations between CHIKV infections and nine meteorological parameters using lagged correlation analysis (Table 3). Several factors exhibited significant lagged effects. Surface pressure (sp) showed the strongest negative correlation with case numbers (*r* = −0.61, *P* < 0.001, lag = 9 days). Temperature-related variables—including 2 m dewpoint temperature (d2m), lake total layer temperature (ltlt), skin temperature (skt), soil temperature (stl1) and 2-m air temperature (t2m)—displayed significant positive correlations (*r* = 0.34–0.57, all *P* < 0.01). Interestingly, soil moisture (swvl1) showed a weak but significant positive lagged correlation (*r* = 0.24, *P* = 0.024, lag = −3 days), whereas total precipitation (tp) and relative humidity (rh) were not significantly associated with case number.

**Table 3.** Lagged association between chikungunya virus infection and meteorological factors.

		Optimal Lag	Correlation		
Climate Variable	Abbreviation	(Days)	Coefficient	95% CI	*P* value
2 meter dewpoint temperature (°C)	d2m	6	0.49	(0.32, 0.65)	< 0.001
Evaporation (mm)	e	14	0.24	(0.03, 0.44)	0.035
Lake total layer temperature (°C)	ltlt	13	0.57	(0.39, 0.71)	< 0.001
Skin temperature (°C)	skt	14	0.41	(0.18, 0.59)	< 0.001
Surface pressure (hPa)	sp	9	−0.61	(−0.73, −0.44)	< 0.001
Soil temperature level 1 (°C)	stl1	14	0.44	(0.25, 0.63)	< 0.001
Soil water volume level 1 (%)	swvl1	−3	0.24	(0.02, 0.44)	0.024
2 meter temperature (°C)	t2m	14	0.34	(0.13, 0.52)	0.002
Total precipitation (mm)	tp	−2	0.18	(−0.04, 0.40)	0.088
Relative humidity (%)	rh	15	−0.19	(−0.41, 0.03)	0.093

Lag correlation analysis was performed to identify delayed associations between daily case counts and meteorological variables. Positive lag values indicate delayed responses of case numbers to climatic variations, while negative lags suggest climate factors leading case fluctuations. Meteorological data were obtained from the ERA5 reanalysis dataset, and chikungunya case data were derived from Guangdong Provincial Center for Disease Control and Prevention.

## DISCUSSION

The CHIKF outbreak in Foshan, a previously non-endemic urban city in China, marks a significant event in the epidemiology of this vector-borne disease. This outbreak not only underscores the growing global risk of emerging infectious diseases but also highlights the unique challenges posed by CHIKV without prior local transmission. The rapid spread of the virus, which resulted in over 6300 confirmed cases within a short period, signals the potential for CHIKV to establish local transmission in areas previously unaffected by the disease. This study, therefore, provides critical insights into the complex interplay between humans, pathogens and environmental conditions in the context of a new geographical setting.

Regarding scale and clinical severity, notable differences were observed across the three outbreaks—Réunion (2024), Yunnan (2019) and Foshan (2025). Since August 2024, widespread transmission of chikungunya disease has been documented in Réunion Island, and more than 47 500 confirmed cases and at least 12 deaths have been reported as of 4 May 2025 [16]. The 2025 Foshan epidemic involved over 19 000 confirmed cases in Guangdong Province, but importantly, no severe or fatal cases have been documented to date, suggesting a predominantly mild clinical profile. The 2019 endemic outbreak in Yunnan, China was much more limited in scale, with just over 100 locally acquired and imported cases, and no reported severe or fatal cases.

One of the key findings of this study is the role of viral mutations in facilitating the rapid spread of CHIKV. The combined mutations E1-A226V, E2-I211T and E2-L210Q, present in the Foshan CHIKV strains, are associated with increased transmission efficiency in *A. albopictus*, which is particularly concerning [17,18]. These mutations are consistent with those found in the Réunion Island lineage, further supporting the idea that such viral adaptations enable CHIKV to thrive in novel environments. Konstantin *et al.* [19] showed that the introduction of either the E1-A226V or E2-I211T point mutation in CHIKV does not result in an increase in viral infectivity. However, when both mutations occur synergistically, they significantly enhance the midgut infectivity of *A. albopictus*. The impact of the E1-A226V mutation on the adaptation of CHIKV to *A. albopictus* is notably more pronounced than that of E2-L210Q. The E2-L210Q mutation, on the other hand, significantly enhances the ability of CHIKV to cause disseminated infections in *A. albopictus* [20]. These studies, however, remain primarily phenotypic in nature, and further investigations are required to explore the underlying mechanistic pathways driving these observations.

The clinical manifestations of CHIKF observed in this cohort also presented challenges in the early diagnosis. While fever, rash and arthralgia are hallmark symptoms of CHIKV infection, the fever, as an initial clinical presentation of the patients in this cohort, was often mild, with adults more frequently exhibiting low- to moderate-grade fever rather than high fever. Over 30% of patients in this cohort initially sought healthcare through departments such as internal medicine, dermatology, pediatrics and rheumatology, rather than in the infectious disease, emergency or fever clinics. This pattern emphasizes the importance of clinicians taking a thorough epidemiological history during the diagnostic process, especially for patients with unexplained fever, rash or joint symptoms [21]. By doing so, the risk of missed diagnoses and subsequent community transmission can be minimized.

Joint involvement was most frequently observed in the ankles, followed by the wrists and knees, whereas the largest-ever-scale CHIKF outbreak in Réunion Island from 2005 to 2006 documented predominant involvement of the metacarpophalangeal joints, knees and wrists [22–25]. Rash distribution differed significantly by age group; adults were more likely to exhibit a widespread rash, including full-body involvement, whereas children tended to have a localized rash limited to the trunk and limbs. The heterogeneity in symptoms, including the presence of isolated rash or joint pain, calls for heightened clinical awareness of non-classical CHIKV presentation [19,21].

Another outbreak, from August 2024 to 4 May 2025, has recorded over 47 500 confirmed chikungunya cases on Réunion island (WHO). The 22 CHIKV strains from Foshan are homologous primarily to those from the 2024 outbreak, underscoring the potential for cross-border transmission, and compared to the Réunion 2005/2006 strains, the 2024 outbreak strain and the Foshan strains carry new mutations: NSP1: N95S and E498K; NSP2: N495S, V516I, P562H and I581V; NSP3: V19A and L410Q; and capsid: T143A. Currently, no studies regarding these new mutation sites and no summaries of clinical manifestations of the 2024–25 outbreak have been reported. Whether these mutations cause the clinical differences remains questionable. The population of Réunion Island is ethnically mixed, with European, Indo-Asian and African descent, whereas the study subjects in this research are all of Asian descent. Thus, we speculate that one of the reasons for the observed clinical differences could be due to the ethnic background, which could result in different host–virus immune responses caused by genetic heterogeneity. Additionally, different lifestyles and work habits could result in various pre-existing joint injuries, which could predispose them to different patterns of joint involvement when a person has CHIKV infection. Another observation among Foshan patients is that adults show more extensive joint involvement than minors. This difference likely arises from distinct immunological responses between pediatric and adult populations, including variations in intensity, cytokine profiles and response dynamics. Studies indicate that pediatric patients exhibit a stronger innate immune response to CHIKV infection when compared to adult patients. This more robust response promotes effective viral clearance and better clinical outcomes, highlighting the importance of considering age-related immune differences in treatment strategies [1,2].

In addition to viral factors, environmental conditions, particularly the climate, played a crucial role in facilitating the outbreak. The statistical analysis of climatic conditions in Foshan revealed a significant increase in days with favorable conditions for vector survival and reproduction in recent years. Notably, 2025 featured the longest continuous period of optimal conditions, which likely contributed to the proliferation of *A. albopictus* [26,27]. Such climate patterns may become more frequent with ongoing climate change, thereby increasing the transmission risk in other regions. Foshan’s experience emphasizes the need to address the interplay between climate change and vector-borne diseases. The result of lag correlation analysis indicate that short-term climatic fluctuations can influence chikungunya transmission. The negative correlation with surface pressure implies that low-pressure conditions may promote *A. albopictus* activity, whereas positive associations with temperature underscore the strong thermal dependence of mosquito behavior, in line with previous findings [28,29]. Moreover, the leading effect of soil moisture indicates that elevated near-surface humidity can rapidly stimulate *A. albopictus* reproduction, consistent with earlier studies [30]. Precipitation and relative humidity showed no significant associations, possibly reflecting urban modification of mosquito habitats. Given the short duration of the outbreak and strong public health interventions, these results should be considered preliminary, providing a clue for future large-scale studies to validate the relationship between climate and case numbers.

From a policy perspective, our findings highlight the need for integrated surveillance and control. Early chikungunya outbreaks in Réunion Island and the Americas showed that delayed detection and reliance on reactive vector control allowed explosive spread and high caseloads [15,31]. Strengthening surveillance in high-risk urban and border areas through entomological monitoring, molecular diagnostics and climate-informed early warning is therefore essential. Diagnostic algorithms should combine rapid RT-PCR for acute cases with serology for convalescent patients to improve differentiation from dengue and other arboviruses. Vector control must be proactive, emphasizing community-based source reduction and targeted interventions supported by the Geographic Information System (GIS) and drone-based tools. These measures together provide a more actionable framework to mitigate future outbreaks in China and other vulnerable regions.

There are several limitations in this study. As patients were recruited during the early stage of the outbreak and only from hospitalized cases at a single center, the findings may be biased toward more severe presentations. Additionally, the clinical data were collected during the acute phase of illness; long-term outcomes such as chronic arthralgia and post-viral sequelae were not systematically assessed. Nonetheless, our research provides a new perspective for CHIKF epidemic prevention and control, and special attention should be paid to patients with atypical symptoms. Timely diagnosis and treatment of the infected patients could put CHIKV infection under control.

This outbreak underscores the importance of a One Health approach, where the health of humans, animals and the environment are considered interconnected. In Foshan, the rapid spread of CHIKV was influenced not only by viral evolution but also by climate conditions. As such, a comprehensive approach that incorporates clinical, virological and environmental data is essential for understanding and mitigating the impact of emerging infectious diseases like CHIKF.

## MATERIALS AND METHODS

### Study design and participants

This observational study recruited 134 patients with CHIKV infection at the beginning of the outbreak. The infection was confirmed by RT‐PCR. They were all admitted to The First People’s Hospital of Foshan for the purpose of quarantine and better clinical management. Upon agreement, we interviewed the participants for their demographic and epidemiological information, the onset date of the illness and the clinical manifestations through a standardized questionnaire. Two independent clinicians double-checked all the data. The study protocol received ethical approval from the Foshan First People’s Hospital Ethics Committee (Approval Nos. 197 and 220), and written informed consent was obtained from all participants prior to their inclusion in the study.

### Assessment of clinical manifestation

Clinical symptoms of the patients were evaluated by clinicians. To determine the joint pain level, children aged ≥5 years and adults were assessed using the Numeric Rating Scale (NRS), ranging from 0 (no pain) to 10 (the highest level of the pain) points; and the Faces Pain Scale-Revised (FPS-R; six facial expressions corresponding to 0, 2, 4, 6, 8 and 10 points) was used for those aged <5 years. All scoring was completed under the guidance of a trained healthcare worker when the patient was relatively calm, and recorded at the first visit. Ultrasound examination on affected joints was performed within 3 days of admission. Through physical examination, we identified the nature and location of the rash on the patient’s body.

### Virological analysis

The viral RNA was extracted and tested using a nucleic acid isolation and detection kit (Da-an Gene Co., Ltd, China). Samples with a CT of ≤38 were considered CHIKV positive. For meta-transcriptomic analysis, RNA was enriched by removing DNA and rRNA, followed by reverse transcription and amplification before sequencing on the MGI‐SEQ2000 platform. Sequencing data were aligned to a reference genome (NC004162), and variants with >40% frequency were analyzed. Phylogenetic analysis was conducted by comparing the complete genome sequences of CHIKV from Foshan and other strains, constructing a tree using IQ-TREE, and assessing mutation sites in MEGA. Viral strains of Foshan cases were randomly sampled after stratifying cases by region. For details, see Supplementary data, Part 1.

### Climate data acquisition and processing

Climate data for Foshan were obtained from the European Centre for Medium-Range Weather Forecasts (ECMWF) ERA5 reanalysis dataset, which provides high-resolution climate data. This dataset includes daily temperature, relative humidity and other atmospheric variables. To align with the study’s temporal focus, the data were processed and aggregated into weekly averages, covering the period from 1 January to 30 June from 2022 to 2025.

### Statistical analysis

Continuous variables were compared using the Mann–Whitney *U*-test and are expressed as median (interquartile range, IQR), whereas categorical variables were analyzed using the chi-squared test or Fisher’s exact test and are represented as counts and percentages. Data analysis was performed using Rstudio (version 4.5.1), and the results were represented graphically using GraphPad Prism (version 10.5.0) and Python (version 3.9).

## Supplementary Material

nwaf529_Supplemental_File

## Data Availability

The data that support the findings of this study originate from the First People’s Hospital of Foshan and are available from the corresponding author upon reasonable request.
