# Supplementary material for: Emerging local chikungunya virus transmission in a major urban area in Southern China: characteristics of clinical manifestations, viral evolution and climatic influences
Source: Natl Sci Rev. 2025 Nov 29;13(4):nwaf529. doi: 10.1093/nsr/nwaf529 (PMC12878321; doi:10.1093/nsr/nwaf529)
Supplement: nwaf529_Supplemental_File [file nwaf529_supplemental_file.docx]

**Content**

[Part 1 Supplementary materials and method 2](#_Toc9378)

[1. Nucleic acid extraction and Real-time polymerase chain reactions 2](#_Toc17097)

[2. Metatranscriptomic library preparation and sequencing 2](#_Toc5730)

[3. Meta-transcriptomic sequencing analysis 2](#_Toc13465)

[4. Phylogenetic analysis 3](#_Toc25948)

[5. Laboratory testing data collection 3](#_Toc28046)

[6. Estimation of R0 3](#_Toc3962)

[Part 2 Supplementary tables 4](#_Toc7262)

[Table S1. Amino Acid Mutations in Chikungunya Virus Strains from the 2025 Foshan Outbreak 4](#_Toc30507)

[Table S2. Laboratory testing of CHIKF patients in different age groups. 9](#_Toc5639)

[Table S3. Multivariable logistic regression analysis of atypical clinical presentation 10](#_Toc20631)

[Table S4. Climatic conditions favorable for vector survival and reproduction in Foshan from 2022 to 2025 11](#_Toc11439)

[Part 3 Supplementary Figures 12](#_Toc16767)

[Figure S1. Pie chart of the severity of joint pain in Chikungunya fever patients across different age. 12](#_Toc25210)

[Figure S2. Rash progression in Chikungunya fever patients (Day 1 to 5). 13](#_Toc4407)

**Part 1 Supplementary materials and method**

**1. Nucleic acid extraction and Real-time polymerase chain reactions**

The serum separated from whole blood sample underwent viral RNA extraction within 4 h using a nucleic acid isolation kit (Da'an Gene Co. Ltd, China). Following this, real-time polymerase chain reaction (RT‐PCR) was performed using the CHIKV viral nucleic acid detection kit (Da'an Gene Co. Ltd, China). The RT-PCR targeted the CHIKV‐specific conserved region and a hostspecific reference gene. The reaction conditions adhered to the manufacturer's specifications, with the cycle threshold (Ct) detection limit set at 38. The receiver operating characteristic (ROC) curve analysis and percentile methods were applied to determine the critical threshold value for kit detection. Samples with Ct values ≤38 were considered positive.

1. **Metatranscriptomic library preparation and sequencing**

The extracted 50 μL nucleic acid was initially digested with DNase I (37 °C, 20 min, MGI, China) to eliminate DNA from the human host and other microorganisms. Next, rRNA was removed through probe hybridization, which involved annealing (95 °C to 22 °C, 0.1 °C /s), followed by RNase H (MGI, China) digestion of the rRNA (37 °C, 30 min) and a final DNase I digestion of the rRNA probes (37 °C, 30 min). This process effectively enriched the viral RNA. The purified RNA was then reverse‐transcribed using random primers, followed by second‐strand synthesis. After end repair and adapter ligation, PCR amplification was conducted using adapter‐specific primers as per the manufacturer's instructions (MGI, China). This unbiased amplification generated PCR products containing viral sequences. The purified products were then used to construct a library (MGI, China), followed by rolling circle amplification to create DNA nanoballs (DNBs). The DNB library was subsequently sequenced using PE100 on the MGI‐SEQ2000 platform.

1. **Meta-transcriptomic sequencing analysis**

The reference genome index was built using BWA (version 0.7.19), and the raw sequencing data was aligned to the reference genome using the bwa mem command. Subsequently, the aligned sequence files were de novo assembled using SPAdes (v4.1.0). Finally, FreeBayes software was employed for variant detection. Mutation sites with a frequency greater than 40% were retained for further mutation analysis.

1. **Phylogenetic analysis**

The full genome sequences of chikungunya virus from 12 patients in Foshan, China, in 2025, along with the full genome sequence of a chikungunya virus strain from Guangzhou, China, in 2024, were compared with 45 full genome sequences of chikungunya virus downloaded from NCBI (https://www.ncbi.nlm.nih.gov/core/ncbi_index/). The above sequences were aligned using MAFFT(v.7.520) software, utilizing the L-INS-i algorithm, where the 5’ and 3’ ends were trimmed and then a maximum likelihood tree was constructed using IQ-TREE v.2.2.0. To reduce the number of sequences prior to temporal analyses, we constructed a maximum likelihood phylogenetic tree with IQ-TREE (v.2.2.0) using the Generalized time-reversible model of Tavare 1986 (GTR) with empirical base frequencies, invariant sites, and invariant sites plus FreeRate model with tree categories (GTR+F+I+G4). Then use ChiPlot (https://www.chiplot.online/) to visualize and enhance the maximum likelihood tree. Finally, perform the analysis of mutation sites in MEGA (v.12.0).

1. **Laboratory testing data collection**

The blood count test and the laboratory biochemical profile were obtained before hospital admission. Other Laboratory and radiology findings were retrieved from the electronic medical charts.

1. **Estimation of R0**

We estimated the basic reproduction number using the Exponential Growth method applied to daily symptom-onset incidence during the initial growth window (6-31 July 2025). The analysis was implemented in the R package R0 and followed procedures consistent with previously published studies[1]. Uncertainty was quantified via parametric bootstrapping (1,000 replicates) to obtain 95% confidence intervals.

**Part 2 Supplementary tables**

**Table S1. Amino Acid Mutations in Chikungunya Virus Strains from the 2025 Foshan Outbreak**

| **Viral Protein** | **Amino Acid Position** | **NC004162/**  **Tanzania**  **/1953** | **HQ846356**  **/China Guangdong/2010** | **MW110476/ChinaYunnan**  **/2019** | **GZ01**  **/2024** | **PV685691**  **/Réunion**  **/2025** | **Foshan**  **Outbreak strains**  **/2025** |
| --- | --- | --- | --- | --- | --- | --- | --- |
| NSP1 | 3 | P | P | P | S | P | P |
|  | 29 | P | P | P | P | S | S |
|  | 34 | P | P | P | S | P | P |
|  | 41 | R | K | R | R | R | R |
|  | 48 | I | I | I | V | I | I |
|  | 75 | D | D | D | D | E | E |
|  | 81 | V | I | V | V | V | V |
|  | 95 | N | N | N | N | S | S |
|  | 128 | T | K | K | T | T | T |
|  | 156 | V | V | V | V | I | I |
|  | 253 | K | K | K | M | K | K |
|  | 351 | T | A | T | T | T | T |
|  | 376 | T | M | M | T | T | T |
|  | 454 | S | S | S | G | S | S |
|  | 473 | S | S | S | R | S | S |
|  | 478 | T | T | T | A | T | T |
|  | 486 | D | D | D | N | D | D |
|  | 487 | A | A | A | A | T | T |
|  | 488 | Q | R | R | Q | K | K |
|  | 491 | R | R | R | Q | R | R |
|  | 498 | E | E | E | E | K | K |
|  | 507 | L | R | R | H | R | R |
| NSP2 | 16 | P | P | P | L | P | P |
|  | 34 | R | R | R | R | C | C |
|  | 54 | S | N | N | S | S | S |
|  | 130 | H | H | Y | H | H | H |
|  | 145 | E | E | D | E | E | E |
|  | 218 | T | T | T | S | T | T |
|  | 273 | Q | Q | Q | L | Q | Q |
|  | 338 | K | K | K | M | K | K |
|  | 352 | P | P | P | S | P | P |
|  | 374 | H | Y | Y | H | Y | Y |
|  | 466 | M | M | M | V | M | M |
|  | 486 | I | I | I | V | I | I |
|  | 495 | N | N | S | N | S | S |
|  | 516 | V | V | V | V | I | I |
|  | 539 | L | S | L | L | L | L |
|  | 562 | P | P | P | P | H | H |
|  | 581 | I | I | I | I | V | V |
|  | 604 | A | A | A | A | V | V |
|  | 756 | V | V | V | I | V | V |
|  | 768 | N | N | N | S | N | N |
|  | 793 | A | V | A | A | A | A |
| NSP3 | 15 | D | D | D | G | D | D |
|  | 19 | A | V | V | V | A | A |
|  | 122 | N | T | T | T | I | I |
|  | 175 | L | I | I | V | I | I |
|  | 176 | L | V | V | I | V | V |
|  | 196 | L | A | A | A | V | V |
|  | 209 | L | A | T | T | T | T |
|  | 213 | L | M | M | V | M | M |
|  | 217 | L | H | H | H | Y | Y |
|  | 283 | M | S | S | N | S | S |
|  | 328 | K | Q | Q | Q | P | P |
|  | 331 | K | A | A | V | A | A |
|  | 332 | K | Q | Q | R | Q | Q |
|  | 335 | K | S | G | S | S | S |
|  | 336 | K | T | T | M | T | T |
|  | 337 | K | I | I | T | A | A |
|  | 338 | K | T | T | T | M | M |
|  | 344 | K | Q | Q | Q | R | R |
|  | 349 | G | V | V | A | V | V |
|  | 355 | G | L | P | P | P | P |
|  | 364 | G | A | A | T | A | A |
|  | 372 | G | D | E | D | D | D |
|  | 384 | G | G | G | E | G | G |
|  | 399 | V | V | V | V | I | I |
|  | 410 | V | L | L | L | Q | Q |
|  | 413 | V | T | T | V | T | T |
|  | 434 | V | L | L | Q | L | L |
|  | 437 | V | V | V | A | V | V |
|  | 439 | V | Q | Q | Q | R | R |
|  | 449 | V | M | M | I | T | T |
|  | 452 | V | Q | Q | R | Q | Q |
|  | 457 | V | T | T | I | T | T |
|  | 458 | V | A | A | T | A | A |
|  | 459 | V | T | T | V | T | T |
|  | 461 | V | P | P | L | L | L |
|  | 462 | V | N | N | S | N | N |
|  | 471 | V | S | S | P | P | P |
|  | 483 | V | N | N | D | S | S |
|  | 484 | V | E | E | D | E | E |
| NSP4 | 43 | P | A | A | L | A | A |
|  | 46 | P | Q | Q | Q | H | H |
|  | 50 | P | T | K | K | K | K |
|  | 55 | P | S | N | S | S | S |
|  | 58 | P | M | M | T | M | M |
|  | 75 | D | A | A | T | T | T |
|  | 82 | D | S | R | R | R | R |
|  | 85 | D | R | G | K | R | R |
|  | 90 | D | S | S | A | S | S |
|  | 101 | N | T | T | V | V | V |
|  | 229 | L | K | K | R | K | K |
|  | 230 | K | K | K | K | N | N |
|  | 235 | A | Q | Q | R | Q | Q |
|  | 254 | S | A | A | T | T | T |
|  | 263 | S | P | P | P | P | P/A* |
|  | 271 | M | K | K | R | K | K |
|  | 280 | M | E | E | D | E | E |
|  | 366 | G | T | T | A | T | T |
|  | 487 | V | M | V | M | M | M |
|  | 497 | V | V | V | V | A | A |
|  | 500 | V | L | L | Q | L | L |
|  | 565 | V | G | E | E | E | E |
|  | 582 | V | V | V | A | V | V |
| CP | 7 | Q | Q | Q | R | Q | Q |
|  | 11 | N | D | N | N | N | N |
|  | 23 | P | S | S | P | P | P |
|  | 27 | V | I | I | V | V | V |
|  | 37 | P | Q | Q | K | Q | Q |
|  | 55 | P | A | A | V | A | A |
|  | 63 | P | R | R | K | R | R |
|  | 73 | P | K | R | K | K | K |
|  | 78 | D | Q | Q | R | Q | Q |
|  | 81 | D | T | T | M | T | T |
|  | 93 | D | A | A | V | A | A |
|  | 122 | N | K | K | K | R | R |
|  | 143 | N | T | T | T | A | A |
| E3 | 33 | P | E | E | K | E | E |
|  | 44 | P | R | R | S | R | R |
|  | 60 | P | H | H | R | H | H |
| E2 | 2 | T | T | T | I | T | T |
|  | 5 | N | N | N | H | N | N |
|  | 74 | P | M | M | M | T | T |
|  | 85 | D | V | V | V | A | A |
|  | 118 | N | S | S | G | G | G |
|  | 142 | N | H | H | Y | H | H |
|  | 149 | N | K | K | R | R | R |
|  | 157 | V | V | V | A | V | V |
|  | 164 | V | T | T | A | T | T |
|  | 194 | L | G | G | S | G | G |
|  | 205 | L | G | S | D | G | G |
|  | 206 | L | S | S | L | S | S |
|  | 207 | L | N | N | S | N | N |
|  | 210 | L | L | L | S | Q | Q |
|  | 221 | L | K | K | R | K | K |
|  | 222 | L | V | V | V | I | I |
|  | 227 | L | A | A | A | V | V |
|  | 246 | A | A | A | A | D | D |
|  | 248 | A | L | L | S | L | L |
|  | 252 | S | Q | K | K | K | K |
|  | 255 | S | I | I | V | I | I |
|  | 264 | S | V | A | V | V | V |
|  | 282 | M | Q | Q | Q | K | K |
|  | 307 | M | Q | Q | R | Q | Q |
|  | 312 | M | M | M | T | T | T |
|  | 313 | M | Y | H | H | H | H |
|  | 317 | I | V | V | I | V | V |
|  | 318 | I | V | V | R | V | V |
|  | 375 | G | T | T | S | S | S |
|  | 384 | G | M | M | V | M | M |
|  | 386 | G | A | A | V | V | V |
|  | 415 | V | I | I | I | L | L |
| 6K | 8 | V | I | I | V | V | V |
|  | 45 | P | T | T | M | T | T |
|  | 47 | P | A | A | T | A | A |
|  | 52 | P | L | M | L | M | M |
|  | 60 | P | S | S | S | N | N |
| E1 | 9 | S | N | N | N | S | S |
|  | 37 | P | T | T | T | I | I |
|  | 72 | P | N | N | S | N | N |
|  | 98 | N | A | A | T | A | A |
|  | 145 | N | T | T | A | T | T |
|  | 196 | L | R | R | K | R | R |
|  | 211 | L | K | E | E | K | K |
|  | 225 | L | A | A | S | A | A |
|  | 226 | L | V | A | A | V | V |
|  | 250 | S | P | S | S | P | P |
|  | 269 | M | V | V | M | V | V |
|  | 284 | M | E | E | D | D | D |
|  | 304 | M | P | P | S | P | P |
|  | 317 | I | I | V | I | V | V |
|  | 321 | I | A | A | T | A | A |
|  | 324 | K | K | K | K | R | R |
|  | 333 | K | M | M | V | M | M |
|  | 348 | G | G | G | G | E | E |
|  | 382 | G | F | P | P | P | P |
|  | 399 | V | V | V | V | I | I |

The yellow-highlighted entries in the table indicate the specific site mutations of the Foshan strains compared with previously reported strains.*:At position 263 of NSP4, all Foshan strains are P except for FSXJ015, which is A. Abbreviations: nsp = Non - Structural Protein; E = Envelope Protein; CP= Capsid Protein.

**Table S2. Laboratory testing of CHIKF patients in different age groups.**

|  | **Overall**(**N=134**) | **＜18 years**(**n=26**) | **18~64years(n=68)** | **≥65 years**(**n=40**) |
| --- | --- | --- | --- | --- |
| **Complete Blood Count** | | | | |
| WBC(10^9^/L) | 5.2[4.2～6.6] | 5.9[4.6～6.8] | 5.4[4.4～6.6] | 4.8[3.4～6.4] |
| NEUT(10^9^/L) | 3.6[2.5～5.2] | 3.26[2.41～5.26] | 3.65[2.77～5.30] | 3.29[2.19～5.06] |
| LYM(10^9^/L) | 0.98[0.67～1.30] | 1.00 [0.82~ 1.74] | 0.99 [0.76~1.33] | 0.72 [0.51~ 1.16] |
| PLT(10^9^/L) | 206.0[167.5～250.3] | 247.0[196.8~ 273.5] | 223.5[183.0~ 257.3] | 162.0[131.5~ 204.0] |
| **Inflammatory Makers** | | | | |
| hsCRP(mg/L) | 10.7[4.0～21.9] | 4.1 [2.1~ 8.7] | 9.4 [4.0~ 18.2] | 19.1 [10.8~ 43.1] |
| PCT(ng/ml) | 0.06[0.03～0.10] | 0.1 [0.08~ 0.12] | 0.1 [0.01~ 0.07] | 0.07[0.04~ 0.11] |
| ESR(mm/h) | 16.0[9.0～30.0] | 9.5 [7.1~ 15.5] | 17.0 [8.3~ 29.8] | 23.5 [14.0~ 34.6] |
| IL-6(pg/ml) | 11.6[6.8～31.2] | 7.8[3.3~1.0] | 11.2[6.6~30.4] | 28.2[11.8~47.5] |
| **Hepatic Enzymes** | | | | |
| ALT(IU/L) | 18.5[13.0～25.0] | 15.0[13.0~23.5] | 20.0[13.0~28.0] | 19.0[14.0~25.0] |
| AST(IU/L) | 22.5[18.0～28.0] | 27.0[21.3~32.8] | 20.0[17.0~26.0] | 25.0[20.3~28.8] |
| **Muscle and Cardiac Enzymes** | | | | |
| LDH(IU/L) | 201.9[181.2～241.0] | 236.6[206.2~275.1] | 196.5[173.5~222.4] | 206.6[189.7~244.2] |
| CK(IU/L) | 74.8[56.0～107.7] | 85.0[62.3~119.9] | 67.8[51.0~91.0] | 88.1[60.4~145.3] |
| CK-MB(IU/L) | 6.9[4.9～9.7] | 10.0[6.5~15.7] | 6.2[4.7~8.0] | 7.8[5.5~9.8] |
| **Urinalysis** | | | | |
| PRO(+,%) | 20(16.7) | 2(9.1) | 6(9.8) | 12(32.4) |

Abbreviations：ALT, alanine aminotransferase; AST, asparagine aminotransaminase; CK, creatine phosphokinase;CK, creatine kinase;CK-MB, creatine kinase-myocardial band; ESR ,erythrocyte sedimentation rate;hsCRP, high-sensitivity C-reactive Protein; IL-6: Interleukin-6;LDH,lactic dehydrogenase; LYM, absolute value of lymphocytes; NEUT, absolute value of Neutrophil; PCT,Procalcitonin; PLT, platelet count; PRO,Protein in Urine;WBC,white blood cell count.

**Table S3. Multivariable logistic regression analysis of atypical clinical presentation**

| Varaible | Atypical N(%) | Typical N(%) | Odds Ratio(95%CI) | *P value* |
| --- | --- | --- | --- | --- |
| **Age (years)** |  |  |  |  |
| <18 | 12 (46.2%) | 14 (53.8%) | Ref. |  |
| 18-64 | 30(44.1%) | 38 (55.9%) | 0.997 (0.392-2.538) | 0.996 |
| >=65 | 19 (47.5%) | 21 (52.5%) | 1.178 (0.356-3.899) | 0.788 |
| **Sex** |  |  |  |  |
| Female | 27 (42.2%) | 37 (57.8%) | Ref. |  |
| Male | 34 (48.6%) | 36 (51.4%) | 1.233 (0.606-2.51) | 0.564 |
| **Comorbidity** |  |  |  |  |
| Hypertension | 14 (42.4%) | 19 (57.6%) | 0.668 (0.243-1.836) | 0.435 |
| Coronary heart disease | 4 (57.1%) | 3 (42.9%) | 1.541 (0.288-8.256) | 0.614 |
| Diabetes | 5 (55.6%) | 4 (44.4%) | 1.555 (0.369-6.552) | 0.547 |

Atypical symptoms included lymphadenopathy, chronic inflammatory rheumatism, articular destruction, bullous dermatosis, hyperpigmentation, stomatitis, xerosis, meningoencephalitis, encephalopathy, seizures, sensorineural abnormalities, Guillain-Barre syndrome, paresis/palsies, neuropathy, nausea, vomiting, abdominal pain , anorexia, diarrhea, hemorrhage, optic neuritis, retinitis, uveitis, myocarditis, pericarditis, heart failure, arrhythmias, cardiomyopathy, fulminant hepatitis, respiratory failure, pneumonia, nephritis, acute renal failure.

**Table S4. Climatic conditions favorable for vector survival and reproduction in Foshan from 2022 to 2025**

| **Year** | **Cumulative Days Meeting Favorable Conditions*** | **Peak Optimal Period**** | **Onset Date of Summer***** | **Avg. Temp since Onset Day (°C)** | **Avg. RH since Onset Day (%)** |
| --- | --- | --- | --- | --- | --- |
| 2022 | 33 | 11 | 2022/4/6 | 22.40 | 77.07 |
| 2023 | 36 | 8 | 2023/4/11 | 22.26 | 73.71 |
| 2024 | 36 | 11 | 2024/3/23 | 23.39 | 84.05 |
| 2025 | 38 | 23 | 2025/3/22 | 22.44 | 83.06 |

Note: *Favorable conditions were defined as days with relative humidity ≥80% and temperature between 26–29°C. **Peak optimal period refers to the maximum number of consecutive days (≥14) meeting both temperature and humidity thresholds. ***The onset date of summer in Foshan is obtained from the Guangdong Meteorological Bureau.

**Part 3 Supplementary Figures**

**Figure S1. Pie chart of the severity of joint pain in Chikungunya fever patients across different age.**


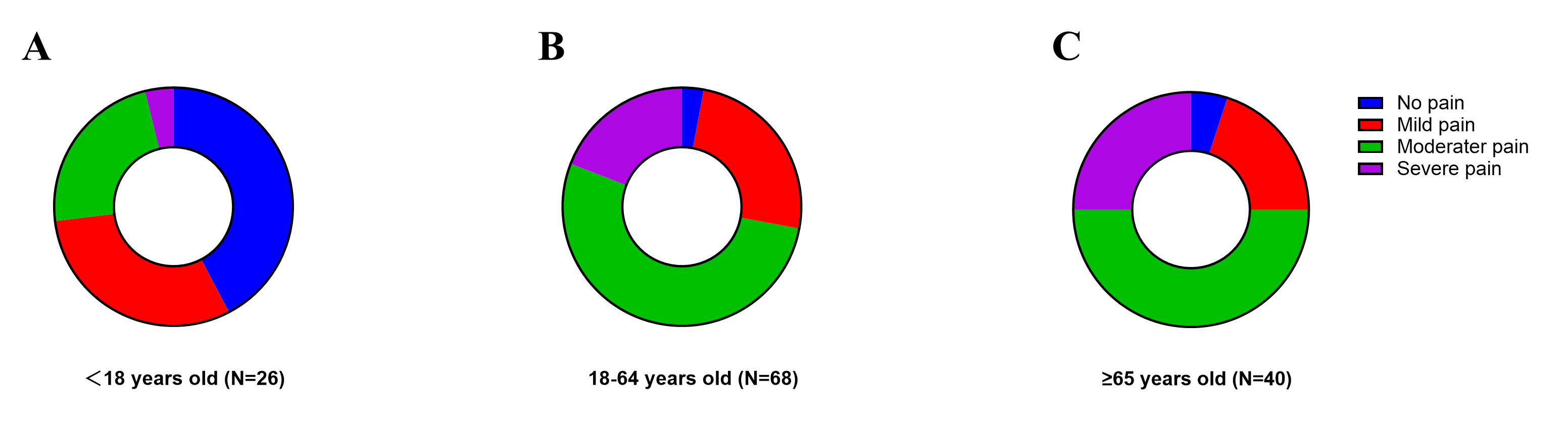


(A) Patients <18 years old (N=26), (B) Patients aged 18-64 years (N=68), and (C) Patients ≥65 years old (N=40). Pain was assessed using the Numeric Rating Scale (NRS) for children ≥5 years and adults, and the Faces Pain Scale-Revised (FPS-R) for children <5 years. Pain levels were classified as: 0 (no pain, blue), 1-3 (mild pain, red), 4-6 (moderate pain, green), and 7-10 (severe pain, purple).

**Figure S2. Rash progression in Chikungunya fever patients (Day 1 to 5).**


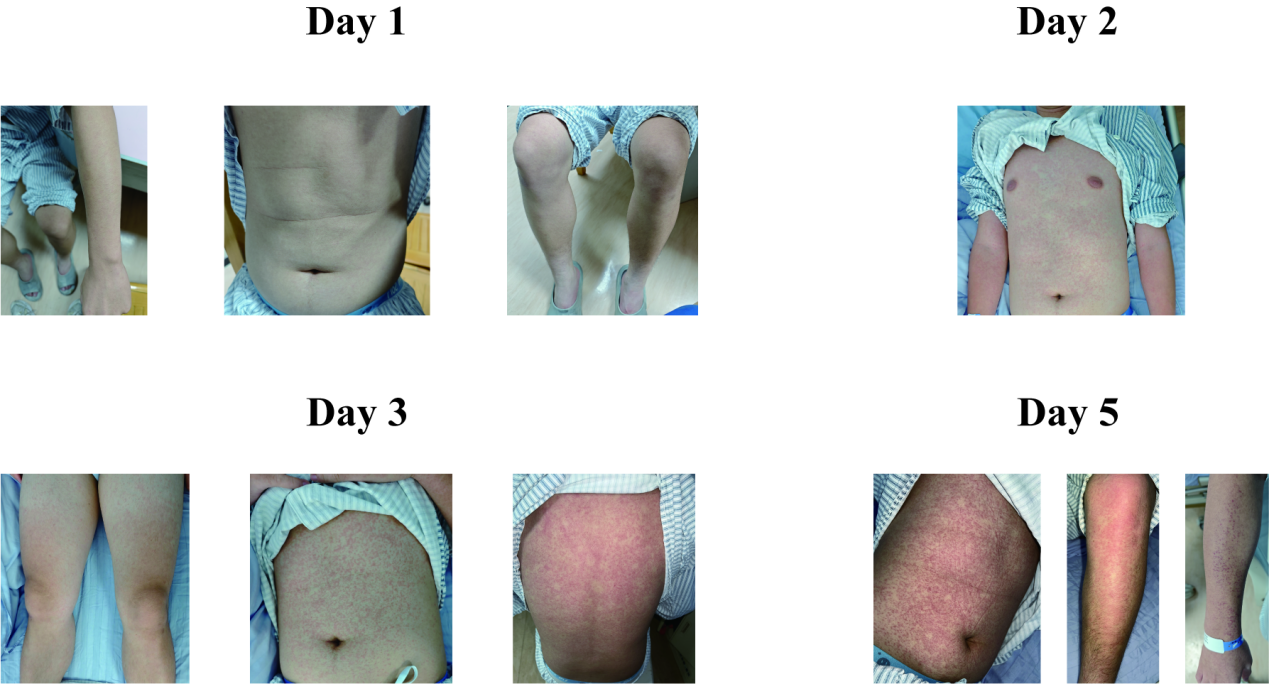


A typical rash of chikungunya virus (CHIKV) infection from one patient is shown. On Day 1 (initial stage), the rash was sparse, localized, symmetric, and maculopapular, primarily appearing on the upper limbs and abdomen. On Day 2 (progression stage), the rash became more pronounced and widespread, especially on the chest and upper body. On Day 3 (advanced stage), the rash continued to spread to the lower body, including the legs, while retaining its maculopapular appearance. On Day 5 (final stage), the rash was more confluent, with some areas showing increased intensity and redness.

Reference

1. Luo, L., et al., *Crucial control measures to contain China's first Delta variant outbreak.* Natl Sci Rev, 2022. **9**(4): p. nwac004.
